# Serum phytosterols associate with T helper 1 cytokine concentration in pregnant women

**DOI:** 10.1002/fsn3.1697

**Published:** 2020-06-10

**Authors:** Yuwei Liu, Wenyun Li, Jiaye Qian, Min Wu, Hongyi Du, Linji Xu, Shuping Liu, Jianping Yi, Gengsheng He

**Affiliations:** ^1^ School of Public Health/Key Laboratory of Public Health Safety of Ministry of Education Fudan University Shanghai China; ^2^ Institute of Reproductive and Child Health School of Public Health Peking University Health Science Center Beijing China; ^3^ Maternal and Child Health Care Hospital Tangshan Municipality Tangshan China

**Keywords:** phytosterol, pregnancy, Th1, Th2, β‐sitosterol

## Abstract

The dietary phytosterols have been demonstrated to modulate CD4^+^ T‐cell polarization in cells, animals, and humans. However, T helper (Th)1/Th2 dichotomy has rarely been correlated with phytosterols during pregnancy. The present study investigated associations between the serum cytokines and serum phytosterols in 100 pregnant women at 34‐ to 37‐week gestation and their offspring. The results showed that serum concentrations of interferon (IFN)‐γ, tumor necrosis factor (TNF)‐α, and total Th1 cytokines were positively associated with serum β‐sitosterol level, adjusting for age, BMI, and serum cholesterol. Serum IFN‐γ and total Th1 cytokine concentrations positively correlated with total phytosterol concentration, controlling age, BMI, and serum cholesterol. Moreover, none of the cytokines measured correlated with phytosterol concentration in the newborns. Our results show that serum Th1 cytokine concentrations, but not Th2 levels, are positively associated with serum phytosterols in pregnant women. These findings implicate that phytosterols modulate Th1/Th2 balance by inducing Th1 secretions in pregnant women.

## Graphical abstract

T helper (Th)1/Th2 dichotomy has been correlated with phytosterols during pregnancy. We found that serum concentrations of interferon (IFN)‐γ, tumor necrosis factor (TNF)‐α, and total Th1 cytokine were positively associated with serum β‐sitosterol level in pregnant women.

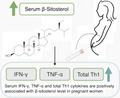

## 1 INTRODUCTION

CD4^+^ T cell differentiates into two major subsets of T helper (Th) cells characterized by differing cytokine profiles. Th1 cells produce interleukin (IL)‐2, interferon (IFN)‐γ, and tumor necrosis factor (TNF)‐α, whereas Th2 cells produce IL‐4, IL‐5, IL‐10, and IL‐13 (Mosmann, Cherwinski, Bond, Giedlin, & Coffman, 1986; Raphael, Nalawade, Eagar, & Forsthuber, 2015). In a healthy situation, Th1 and Th2 cell polarization is well balanced, while a Th1 domination related to autoimmune and inflammatory diseases with a Th2 bias associated with allergies and asthma (Kidd, 2003). Meanwhile, a Th1 to Th2 shift was known accountable for a successful birth outcome in normal human pregnancy (Reinhard, Noll, Schlebusch, Mallmann, & Ruecker, 1998; Sykes, MacIntyre, Yap, Teoh, & Bennett, 2012).

Phytosterols have been popular as health supplements in improving cardiovascular health by their cholesterol‐lowering activities (Abumweis, Barake, & Jones, 2008). The general recommendation is to take 2–3 g phytosterols per day to reduce plasma low‐density lipoprotein cholesterol (Laitinen & Gylling, 2012). In recent years, several reports arouse the interests in phytosterols modulating the immune function, especially for the Th1/Th2 balance. β‐Sitosterol and its glucoside have been found effective in increasing the concentrations of the Th1 cytokines in vitro (Bouic et al., 1996; Desai et al., 2009). Soybean‐derived plant sterol mixtures have also been observed to weaken TNF‐α and IL‐6 production in ApoE^−/−^ mice (Yeganeh, HayGlass, Moghadasian, & Nashed, 2005). In addition, a previous study has reported sitosterol/sitosterol–glucoside mixtures decrease stress‐induced IL‐6 production in marathon runners (Bouic et al., 1999). A mixture of campesterol and β‐sitosterol has been suggested to increase IL‐10 concentration, with a concomitant significant reduction of IL‐1β in postmenopausal women (Alvarez‐Sala et al., 2018). However, no association was detected between sitosterol (or campesterol) and serum TNF‐α (or IL‐6) concentrations in subjects with impaired glucose tolerance (Hallikainen et al., 2007). In general, the correlation between serum phytosterols and cytokines remains unclear in humans. Moreover, the role of phytosterols in mediating Th1/Th2 balance is yet to uncover in the pregnant women and newborns.

In this study, we proposed a hypothesis that phytosterols might be involved in Th1/Th2 dichotomy during pregnancy and in the newborns. To test this hypothesis, we investigated the associations between the serum phytosterol and cytokine concentrations in pregnant women, as well as their offspring. Results of this study will benefit the understanding of phytosterols in mediating maternal immune function and potentially lead to the development of clinical plant sterol advisory during pregnancy.

## 2 MATERIALS AND METHODS

### 2.1 Study design and participants

This research was a prospective cohort study of 924 pregnant women recruited at the 1st trimester (12–15 gestational weeks) in Maternal and Child Health Care Hospital (Tangshan, Hebei, China) from September 2013 to June 2014. Clinical examinations occurred every trimester (1st, 12–15 gestational weeks; 2nd, 24–28 weeks; 3rd, 34–37 weeks) with written consent. We included the participants who were 20–40 years old without selected diseases including preconception diabetes, hypertension, heart disease, chronic renal disease, systemic lupus erythematosus, hypothyroidism, mental disease, and severe anemia.

### 2.2 Data collection

Demographic characteristics and health information were collected by face‐to‐face interview using a questionnaire including prepregnancy weight, height, and family heredity at the baseline visit (at 12–15 weeks). We included fasting blood samples collected from 100 mothers at the baseline visit and the 3rd trimester (at 34–37 weeks), which were available for a secondary post hoc analysis of both serum sterol and cytokine. The corresponding cord blood of their newborns was collected during childbirth. GDM screening was performed at the 2nd‐trimester (24–28 weeks) gestation with an oral glucose tolerance test (OGTT). GDM was diagnosed based on the criteria from International Association of Diabetes and Pregnancy Study Groups (IADPSG, fasting plasma glucose 5.1 mmol/L and/or 1‐hr glucose 10.0 mmol/L and/or 2‐hr 8.5 mmol/L glucose) (International Association of Diabetes & Pregnancy Study Groups Consensus Panel, 2010). Maternal outcomes were obtained from the hospital's database. This study was approved by the Research Ethics Committee of School of Public Health, Fudan University, China (IRB#2013‐07‐0460), and all participants approved the procedures by written consent.

### 2.3 Clinical sample measurements

Plasma total cholesterol (TC), high‐density lipoprotein cholesterol (HDL–C), total triacylglycerols (TG), and glucose were determined by the AU5800 Clinical Chemistry Analyzer (Beckman Coulter). Plasma insulin was measured by the UniCel DxI 800 immunoassay analyzer (Beckman Coulter).

### 2.4 Determination of noncholesterol steroids in serum

Serum noncholesterol steroids from mothers or newborns' cord blood were analyzed using a method modified from which we previously described (Liu et al., 2015, 2017). In brief, total serum lipids were extracted using a mixture of chloroform and methanol (2:1, vol/vol) with the addition of 5α‐cholestane as an internal standard, followed by saponification. Steroids in the nonsaponified fraction were converted to its TMS‐ether derivatives by a commercial TMS reagent (Sigma). The analysis of steroids TMS‐ether derivative was performed in a capillary DB‐5ms (30 m × 0.25 µm, i.d. = 0.25 mm) column (Agilent) using a GC‐2010 Plus High‐end Gas Chromatograph (Shimadzu) equipped with a flame ionization detector. Serum noncholesterol steroids were calculated according to the amount of internal standard 5α‐cholestane added.

### 2.5 Determination of cytokines in serum

Cytokines in serum samples were measured by a fluorescent suspension array system Bio‐Plex 200 (Bio‐Rad) using a Bio‐Plex Pro Human Cytokine 17‐Plex Panel (#M5000031YV) according to the manufacturer's protocol. Bio‐Plex 200 system was validated by a Validation Kit (#171203001) within 1 week of assays and calibrated by a Calibration Kit (#171203060) on each assay day. The quantifications of IL‐2, IL‐4, IL‐5, IL‐6, IL‐10, IL‐13, IFN‐γ, and TNF‐α were included in this study.

### 2.6 Statistics

Baseline clinical characteristics, serum sterols, and cytokine concentrations are presented as mean ± *SD*, frequencies (*n*%), or median (interquartile range) for non‐normally distributed variables. We modeled associations between serum noncholesterol steroids and serum cytokine concentrations in the mothers as well as their newborns using a generalized linear model. The analysis was implemented by the log link function in a GENMOD procedure using SAS studio (University Edition). Significance was defined as *p* value < .05.

## 3 RESULTS

### 3.1 Clinical characteristics

Table 1 summarizes the biomedical characteristics and pregnancy outcomes for all participants in this study. The mean age of participants was 28.1 ± 3.2 years with a median BMI of 21.8 kg/m^2^ at baseline (12–15 weeks). The mean fasting total cholesterol (TC) and high‐density lipoprotein cholesterol (HDL‐C) concentrations of participants were 6.4 ± 1.4 and 1.6 ± 0.4 mmol/L at 34–37 weeks, respectively. The median (interquartile range) of fasting glucose and insulin concentrations was 4.1 (3.8–4.4) mmol/L and 7.5 (5.0–10.7) mIU/L at 34–37 weeks. One‐fifth (20/100) of the pregnant women were diagnosed with GDM at 24‐ to 28‐week gestation, with 17 newborns classified as macrosomia.

**TABLE 1:** Clinical characteristics of participants

	*N*	Mean ± *SD* or Median (interquartile range)^a^
Maternal characteristics (at baseline: 12–15 weeks)		
Age (years)	100	28.1 ± 3.2
BMI (kg/m^2^)	100	21.8 (20.2–24.0)
Family history of diabetes (%)	100	24
Serum lipids (at 34–37 weeks)		
TC (mmol/L)	99	6.4 ± 1.4
HDL‐C (mmol/L)	99	1.6 ± 0.4
TG (mmol/L)	99	3.5 (2.7–4.4)
Glycemic outcomes (at 34–37 weeks)		
Glucose (mmol/L)	98	4.1 (3.8–4.4)
Insulin (mIU/L)	100	7.5 (5.0–10.7)
Pregnancy outcomes (during pregnancy/at delivery)		
GDM (%)	100	20
Macrosomia (%)	100	17

Abbreviations: BMI, body mass index; GDM, gestational diabetes mellitus; HDL‐C, high‐density lipoprotein cholesterol; TC, total cholesterol; TG, total triglyceride.

^a^ Values are mean ± standard deviation or median (interquartile range) or *n* (%).

### 3.2 Serum cytokine and steroid concentrations in pregnant women and newborns

Serum cytokine concentrations of mothers at 34‐ to 37‐week gestation and their newborns are presented in Table 2. Serum total Th1 and Th2 concentrations were dramatically decreased (*p* value = .0001 and .0001, respectively) in the cord blood (5.37 and 10.84 pg/ml, respectively) compared with those in the mothers of late pregnancy (57.51 and 100.51 pg/ml, respectively). Specifically, the concentrations of serum IL‐6, IL‐10, IL‐12, and IFN‐γ in the cord blood noticeably decreased to 8.98, 3.03, 0.70, and 1.71 pg/ml, respectively (Table 2). Table 3 shows serum noncholesterol steroid concentrations of the mothers at 34–37 weeks and their newborns. We observed declining serum total phytosterols in the cord blood (7.50 μmol/L) compared with those in the mothers of late pregnancy (18.22 μmol/L). Desmosterol and lathosterol are cholesterol precursors in serum (Kempen, Glatz, Gevers Leuven, van der Voort, & Katan, 1988; Nissinen, Gylling, & Miettinen, 2008). To fully understand the noncholesterol steroid profile in the mothers and newborns, we also measured the serum desmosterol and lathosterol. We found the concentrations of desmosterol (5.74 vs. 10.36 μmol/L, mothers vs. newborns, respectively, *p* value = .0001) and lathosterol (8.27 vs. 17.68 μmol/L, mothers vs. newborns, respectively, *p* value = .0001) were elevated in the cord blood than those in the mothers of late pregnancy (Table 3).

**TABLE 2:** Serum cytokines of pregnant women and newborns

Cytokines (pg/ml)	Pregnant women (at 34–37 weeks)		Newborns (cord serum)		*p* value*
	*N*	Mean ± *SEM* ^a^	*N*	Mean ± *SEM*	
IL‐2	99	3.55 ± 1.43	56	3.05 ± 0.65	.5469
IL‐4	99	1.01 ± 0.10	99	1.45 ± 0.12	.0067
IL‐5	59	0.36 ± 0.20	47	0.18 ± 0.16	.1158
IL‐6	98	31.73 ± 10.87	98	8.98 ± 3.61	.0001
IL‐10	99	66.60 ± 48.52	99	3.03 ± 0.51	.0003
IL‐12	99	22.32 ± 14.88	99	0.70 ± 0.29	.0001
IL‐13	99	0.80 ± 0.30	99	0.23 ± 0.07	.3837
IFN‐γ	99	24.06 ± 11.08	99	1.71 ± 0.63	.0038
TNF‐α	92	7.58 ± 1.32	95	2.97 ± 0.68	.0035
Total Th1	92	57.51 ± 19.79	95	5.37 ± 1.27	.0001
Total Th2	98	100.51 ± 49.30	98	10.84 ± 3.68	.0001

Abbreviations: IFN‐γ, interferon‐γ; IL, interleukin; Th1, T helper 1; Th2, T helper 2; TNF‐α, tumor necrosis factor‐α.

^a^ Values are mean ± standard error of the mean.

* *p* value: Wilcoxon rank sum test of serum cytokine concentrations (pg/ml) between groups; significance was defined as *p* value < .05.

**TABLE 3:** Serum noncholesterol steroids of pregnant women and newborns

Steroids (μmol/L)	Pregnant women (at 34–37 weeks)		Newborns (cord serum)		*p* value*
	*N*	Mean ± *SEM* ^a^	*N*	Mean ± *SEM*	
Campesterol	99	9.07 ± 0.10	99	2.93 ± 0.09	.0001
Stigmasterol	99	2.26 ± 0.08	95	1.14 ± 0.07	.0043
β‐Sitosterol	99	6.89 ± 0.19	99	3.43 ± 0.08	.0011
Total phytosterols	99	18.22 ± 0.32	99	7.50 ± 0.14	.0001
Desmosterol	98	5.74 ± 0.23	99	10.36 ± 0.42	.0001
Lathosterol	99	8.27 ± 0.24	97	17.68 ± 0.44	.0001

^a^ Values are mean ± standard error of the mean.

* *p* value: Wilcoxon rank sum test of serum steroid concentrations (μmol/L) between groups; significance was defined as *p* value < .05.

### 3.3 Maternal cytokines are associated with phytosterol concentrations in serum

To evaluate how phytosterols modulate CD4^+^ T‐cell differentiation and subsequent Th1/Th2 profiles, we correlated serum cytokines with phytosterol concentrations in pregnant women at 34‐ to 37‐week gestation, as well as their corresponding newborns. In pregnant women, serum IFN‐γ positively correlated with β‐sitosterol (*β* = 3.22, *p* value = .0005) and total phytosterol concentration (*β* = 0.53, *p* value = .0002), whereas TNF‐α only significantly correlated with β‐sitosterol (*β* = 0.42, *p* value = .0005), adjusting for age, BMI, and serum cholesterol (Table 4). Also, serum TNF‐α was marginally associated with serum campesterol level (*β* = 1.63, *p* value = .0547). In addition, total Th1 cytokine concentration was associated with both β‐sitosterol (*β* = 3.27, *p* value = .0006) and total phytosterol concentration (*β* = 0.67, *p* value = .0234), controlling age, BMI, and serum cholesterol (Table 4). No serum Th1 cytokine was associated with stigmasterol level after correction for age, BMI, and serum cholesterol (Table 4). Moreover, none of the Th2 cytokine investigated correlated with phytosterol concentration after adjusting for age, BMI, and serum cholesterol (data not shown). Furthermore, no association was observed between serum cytokines and phytosterols in the cord blood of newborns (data not shown).

**TABLE 4:** Serum phytosterols associated with cytokines in pregnant women

Phytosterols (μmol/L)	Cytokines (pg/ml)	*N*	*β*	*SE*	*p* value*
Campesterol	IL‐2	99	0.23	0.24	.3117
	IFN‐γ	99	1.71	0.77	.2478
	TNF‐α	92	1.63	0.85	.0547
	Total Th1	92	2.87	1.07	.1844
Stigmasterol	IL‐2	99	0.24	0.27	.4537
	IFN‐γ	99	0.49	0.35	.2388
	TNF‐α	92	0.31	0.55	.6035
	Total Th1	92	0.63	0.65	.4429
β‐Sitosterol	IL‐2	99	0.33	0.23	.2008
	IFN‐γ	99	3.22	0.22	.0005
	TNF‐α	92	0.42	0.12	.0005
	Total Th1	92	3.27	0.95	.0006
Total phytosterols	IL‐2	99	0.21	0.16	.1991
	IFN‐γ	99	0.53	0.14	.0002
	TNF‐α	92	0.16	0.08	.0629
	Total Th1	92	0.67	0.29	.0234

Abbreviations: IFN‐γ, interferon‐γ; IL‐2, interleukin‐2; Th1, T helper 1; TNF‐α, tumor necrosis factor‐α.

* *p* value: associations between serum cytokine (pg/ml) and phytosterol (μmol/L) concentrations in the mothers using a generalized linear model by the log link function in GENMOD procedure, adjusted for age, BMI, and serum cholesterol. Significance was defined as *p* value < .05.

## 4 DISCUSSION

We have observed maternal serum Th1 cytokines, but not Th2, are positively associated with serum phytosterol levels. Our findings suggest that phytosterols, specifically β‐sitosterol, modulate Th1/Th2 balance by inducing Th1 secretions in pregnant women.

Moreover, we have found a decreased serum cytokine and phytosterol concentration in the cord blood compared with those in the mothers.

This study is the first time to uncover a potential regulatory role of phytosterol on CD4^+^ T‐cell polarization in pregnant women from a perspective of Th1/Th2 profile. As mentioned above, various studies have demonstrated the correlations between phytosterol and CD4^+^ T‐cell polarization in cells, animals, and humans (Alvarez‐Sala et al., 2018; Bouic et al., 1996, 1999; Desai et al., 2009; Yeganeh et al., 2005). However, maternal Th1/Th2 balance has rarely been correlated with phytosterols. Blood immune status was documented to be associated with weight and age (Flynn, Markofski, & Carrillo, 2019; Gong et al., 2014), and therefore, we matched BMI and age for analysis. To eliminate the effect of differing cholesterol levels in the mothers and newborns, we also controlled the serum concentrations of cholesterol in the analysis. Our data from all the mothers indicate that serum concentration of IFN‐γ and TNF‐α levels is positively associated with β‐sitosterol concentration at 34‐ to 37‐week gestation, implicating β‐sitosterol could induce CD4^+^ T‐cell polarization to a Th1 pole, which subsequently leads to a Th2 to Th1 shift in the blood cytokine profile. Our results are partially in agreement with the previous reports that β‐sitosterol could increase IFN‐γ production in human PBMCs (Brull, Mensink, van den Hurk, Duijvestijn, & Plat, 2010) and induce TNF‐α secretion from macrophages (Kurano et al., 2011).

We report a lower cord serum cytokine and phytosterol concentrations in the newborns than those in their mothers, which are in line with the previous findings on mothers and infants (Correani et al., 2018; Miettinen, Rono, Koivusalo, Eriksson, & Gylling, 2018; Werlang et al., 2018; Woo et al., 2013). However, we have not found correlations between the cord serum cytokine and phytosterol concentrations. It has been suggested that phytosterols could cross the human placenta in a relatively easy effort (Correani et al., 2018), and cord blood cytokines play vital role in evaluating the immune and neural development of newborns (Deverman & Patterson, 2009; Sandberg et al., 2009). Future research is warranted to elucidate the impact of phytosterols on the neonatal cytokines.

A Th1 to Th2 shift in the maternal immune system has been believed as an important prerequisite for a successful pregnancy (Saito, Nakashima, Shima, & Ito, 2010). Our data suggest that phytosterols favor a reverse shift of Th2 to Th1 during pregnancy. Indeed, phytosterol is pervasively used as a therapeutic option to reduce blood cholesterol in populations. Are phytosterol supplements safe for pregnant women? To date, limited information is available in terms of clinical safety of consuming phytosterols during pregnancy. Our results of phytosterol‐associated Th1 shifts might address the maternal safety concern of phytosterol supplements. To our best knowledge, phytosterols are not commonly recommended for reducing cholesterol in pregnant women due to the unestablished safety rules (Correani et al., 2018). Further investigations are warranted to understand the mechanism of phytosterol modulating the maternal immune balance and more importantly its effects on pregnancy outcomes.

A few limitations should be acknowledged when interpreting the results of this study. First, this is a secondary post hoc study of blood samples available for phytosterol and cytokine analysis with no formal power calculation, which might undermine possible correlations between phytosterol and cytokine concentrations. Future phytosterol intervention study is needed to confirm our results. Second, we only managed to record the data on serum IL‐5 of mothers and IL‐2, IL‐5 of newborns for approximately half of the participants because of the low sensitivity of equipment and kits. These lost samples might have affected the result quality.

## 5 CONCLUSION

In summary, we have observed that serum Th1 cytokine concentrations, but not Th2 levels, are positively associated with serum phytosterols in pregnant women. Specifically, serum concentrations of IFN‐γ, TNF‐α, and total Th1 cytokine are positively associated with β‐sitosterol level. Our results of phytosterol‐facilitated Th1 shifts at 34‐ to 37‐week gestation provide insights into the mechanisms of phytosterol mediating immune responses in pregnant women, and might address the maternal safety concern of phytosterol supplements. Follow‐up mechanistic studies are needed to reveal the precise effect size and molecular mechanism. The ultimate goal is to transfer these findings into dietary phytosterol advisory and potentially improve the maternal outcomes.

## CONFLICT OF INTEREST

The authors declare no conflict of interest.

## ETHICAL APPROVAL

This study was approved by the Research Ethic Committee of School of Public Health, Fudan University, China (IRB#2013‐07‐0460), and all participants approved the procedures by written consent.
